# Supplementary material for: A Quadruplex RT-qPCR for the Detection of African Swine Fever Virus, Classical Swine Fever Virus, Porcine Reproductive and Respiratory Syndrome Virus, and Porcine Pseudorabies Virus
Source: Animals (Basel). 2024 Dec 9;14(23):3551. doi: 10.3390/ani14233551 (PMC11640363; doi:10.3390/ani14233551)
Supplement: Supplementary file 1 [file animals-14-03551-s001.zip › animals-3283402-supplementary.pdf]

Supplementary Materials

Table S1. The information on ASFV strains

Strain	GenBank No.	Country	Collection date	Submitted date	Genotype
MAD/1/98	AF270706.1	Mozambia	2003	2016	II
SPEC265	AF270710.1	Mozambia	2003	2016	VI
MOZ/94/1	AF270711.1	Mozambia	2003	2016	VI
Tengani	AF301541.1	Malawi	2003	2016	V
CAM/4/85	AF301545.1	Cameroon	2003	2016	I
BUR/1/84	AF449463.1	Uganda	2003	2016	X
ZAR85	AF449465.1	Spain	2003	2016	I
UGA/1/95	AF449475.1	Uganda	2003	2016	IX
UGA/3/95	AF449476.1	Uganda	2003	2016	X
RSA/1/99/W	AF449477.1	South Africa	2003	2016	IV
NIG-2	AF504884.1	Nigeria	2003	2016	I
BOT/1/99	AF504886.1	Botswana	2003	2016	III
GHA/1/00	AF504888.1	Ghana	2003	2016	I
MOZ-60/98	AY274455.1	Mozambia	2004	2016	II
KAB/62	AY351522.1	Zambia	2005	2016	XI
SUM/1411	AY351542.1	Zambia	2005	2016	XIII
MZI/921	AY351543.1	Malawi	2005	2016	XII
THY/901	AY351545.1	Malawi	2005	2016	VIII
MWHOG/3	AY351549.1	Malawi	2005	2016	X
NYA/12	AY351555.1	Zambia	2005	2016	XIV
LUS93/1	AY351563.1	Zambia	2005	2016	II
TAN/2003/1	AY494550.1	Tanzania	2005	2016	XVI
TAN/1/01	AY494552.1	Tanzania	2005	2016	XV
SPEC/154	DQ250113.1	Botswana	2007	2016	VII
SPEC/245	DQ250117.1	South Africa	2007	2016	XXII
ZIM/92/1	DQ250119.1	Zimbabwe	2007	2016	XVII
SPEC/260	DQ250121.1	South Africa	2007	2016	VII
NAM/1/95	DQ250122.1	Namibia	2007	2016	XVIII
RSA/1/95	DQ250123.1	South Africa	2007	2016	XX
RSA/1/96	DQ250125.1	South Africa	2007	2016	XXI
RSA/2/96	DQ250126.1	South Africa	2007	2016	XIX
Ug03H.1	FJ154428.1	Uganda	2009	2016	IX
Ken06.B3	FJ154436.1	Spain	2009	2016	IX
Kat67	FJ174377.1	Congo	2009	2016	I
Ang72	FJ174378.1	Angola	2009	2016	I
Nig01	FJ174382.1	Nigeria	2009	2016	I
Ken05/Tk5	HM745257.1	Kenya	2011	2016	X
Tan 2011/01	JX310038.1	Tanzania	2011	2013	II
KEN 2001/2	JX524215.1	Kenya	2001	2020	IX

TAN/13/Moshi	KF706360.1	Tanzania	2013	2015	X
ETH/3	KT795360.1	Ethiopia	2011	2016	XXIII
MOZ_16/2006	KY353995.1	Mozambia	2006	2017	XXIV
ASFV-SY18	MH766894.1	China	2018	2018	II
China/2018/AnhuiXCGQ	MK128995.1	China	2018	2021	II
Pig/HLJ/2018	MK333180.1	China	2018	2019	II
DB/LN/2018	MK333181.1	China	2018	2019	II
ASFV-wbBS01	MK645909.1	China	2018	2021	II
CN/2019/InnerMongolia-AES01	MK940252.1	China	2019	2021	II
ASFV/pig/China/CAS19-01/2019	MN172368.1	China	2019	2020	II
ASFV Wuhan 2019-1	MN393476.1	China	2019	2020	II
ASFV Wuhan 2019-2	MN393477.1	China	2019	2020	II

Table S2. The information on CSFV strains

Strain	GenBank No.	Country	Collection date	Submitted date	Genotype
Eystrup	AF326963.1	Switzerland	/	19-Dec-2003	1.1
CSFV/1.1/dp/CSF0382	HM237795.1	Germany	/	18-Oct-2010	1.1
CSFV-GZ-2009	HQ380231.1	China	2009	5-Apr-2011	1.1
CSFV-PK15C-NG79-11	KC503764.1	India	Sep-2011	2-Jun-2015	1.1
Koslov clone Kos_4aa	KF977610.1	Denmark	1-Jan-2013	20-Nov-2014	1.1
Alfort A19	U90951.1	France	/	26-Mar-2097	1.1
CS	AF099102.3	Russia	/	3-Oct-2000	1.2
BRESCIAX	AY578687.1	USA	2001	31-Mar-2005	1.2
0406/CH/01/TWN	AY568569.1	Taiwan, China	/	1-Jan-2005	2
YC11WB	KC149990.1	Korea	2011	26-Apr-2013	2
SXYL2006	GQ122383.1	China	Mar-2006	15-Jun-2009	2.1
GD53/2011	KP343640.1	China	2011	1-May-2016	2.1
HL18-462	MT799517.1	China	2016/2018	16-May-2021	2.1
HL18-490	MT799518.1	China	2016/2018	16-May-2021	2.1
Paderborn	AY072924.1	Denmark	/	7-Feb-2002	2.1a
uN23/2013	KP233071.1	China	Jun-2013	30-Jul-2015	2.1b
CSFV-JXNC01-2015	KX064281.1	China	Mar-2015	12-Dec-2016	2.1b
HNSD-2012	JX218094.1	China	May-2012	13-Mar-2013	2.1c
HNLY-2011	JX262391.1	China	Mar-2011	13-Mar-2013	2.1c
GXF29/2013	KP233070.1	China	Jun-2013	23-Jun-2015	2.1c
JSZL	KT119352.1	China	Dec-2014	27-Aug-2015	2.1d
HeN1505	KU556758.1	China	May-2015	24-May-2016	2.1d
SDSG1410	MF150645.1	China	2014	17-Mar-2018	2.1d
39	AF407339.1	China	/	6-Sep-2001	2.2
CSFV_IND/UK/LAL-290	KC851953.1	India	5-Jan-2012	21-May-2014	2.2
CSFV/2.3/wb/CSF1046	GU233733.1	Germany	2009	18-Oct-2010	2.3
CSFV/2.3/wb/CSF1045	GU233734.1	Germany	2009	18-Oct-2010	2.3
Alfort/Tuebingen	J04358.2	Germany	/	23-Nov-2005	2.3
CSFV/2.3/SRB/6168/2006	KY849594.1	Serbia	4-Sep-2006	24-Dec-2019	2.3
CSFV/2.3/dp/CSF864	HQ148062.1	Germany	2007	12-Jul-2011	2.3d
JJ9811	KF669877.1	Korea	1998	9-Mar-2016	3.2
94.4/IL/94/TWN	AY646427.1	Taiwan, China	1994	29-Oct-2007	3.4
P97	L49347.1	/	/	2-Aug-2006	3.4

Table S3. The information on PRRSV strains

Strain	GenBank No.	Country	Collection date	Submitted date	Genotype
FJZ03	KP860909.1	China	/	19-Aug-2015	Lineage 1
HNhx	KX766379.1	China	2016	23-Oct-2016	Lineage 1
HNjz15	KT945017.1	China	2015	3-May-2016	Lineage 1
MN184A	DQ176019.1	USA	/	2-Mar-2022	Lineage 1
FJFS	KP998476.1	China	/	19-Aug-2015	Lineage 3
GM2	JN662424.1	China	Jan-2011	13-Aug-2012	Lineage 3
HNyc15	KT945018.1	China	2015	10-May-2016	Lineage 3
QYYZ	JQ308798.1	China	16-Jan-2011	13-Aug-2012	Lineage 3
BJ-4	AF331831.1	China	/	14-Jul-2016	Lineage 5
HN1	AY457635.1	China	/	26-Jul-2016	Lineage 5
RespPRRS MLV	AF066183.1	USA	/	26-Apr-2019	Lineage 5
VR-2332	DQ217415.1	USA	/	31-Aug-2007	Lineage 5
CH-1R	EU807840.1	China	/	26-Jul-2016	Lineage 8
GX1003	JX912249.1	China	2010	12-Jan-2013	Lineage 8
CH-1a	AY032626.1	China	/	22-Jul-2016	Lineage 8
HeNan-A9	KJ546412.1	China	4-Jul-2013	25-May-2014	Lineage 8
HH08	JX679179.1	China	2011	18-Nov-2012	Lineage 8
NADC30	JN654459.1	USA	2008	12-Oct-2012	NADC30-Like
SD217-1705	MT093771.1	China	May-2017	20-Feb-2022	NADC30-Like
NL1207	MZ399800.1	China	2019	14-Aug-2022	NADC30-Like
IA/2014/NADC34	MF326985.1	USA	2014	2-Dec-2017	NADC34-Like
GD-H1	ON691479.1	China	Oct-2021	6-Dec-2022	NADC34-Like
Amervac PRRS	GU067771.1	China	/	24-Jul-2016	Amervac-Like
DK-2003-7-2	KC862572.1	Denmark	2003	13-Dec-2013	NMEU09-1-Like
HeB3	MN927227.1	China	2018	20-Feb-2021	BJEU06-1-Like
BJEU06-1	GU047344.1	China	2006	24-Jul-2016	BJEU06-1-Like
NL/GD-1-3/2015	MK404232.1	Netherlands	2015	6-Jul-2019	BJEU06-1-Like
HK3	KF287129.1	Hong Kong, China	1-Jan-2003	10-Apr-2014	HKEU16-Like
HKEU16	EU076704.1	Hong Kong, China	/	26-Jul-2016	HKEU16-Like
HK5	KF287130.1	Hong Kong, China	1-Jan-2004	10-Apr-2014	HKEU16-Like
HK10	KF287131.1	Hong Kong, China	1-Jan-2004	10-Apr-2014	HKEU16-Like

Table S4. The information on PRV strains

Strain	GenBank No.	Country	Collection date	Submitted date	Genotype
Bartha	JF797217.1	Hungary	/	11-Apr-2011	II
Kaplan	KJ717942.1	Hungary	/	11-Apr-2014	II
NIA3	KU900059.1	United Kingdom	1970	11-Mar-2016	II
HUB17	MT949537.1	China	Aug-2020	01-Sep-2020	I
Fa	KM189913.1	China	06-Aug-2012	13-Jul-2014	I
Ea	KU315430.1	China	1990	17-Dec-2015	I
Becker	JF797219.1	USA	/	11-Apr-2011	II
SC	KT809429.1	China	1986	23-Sep-2015	I
HN1201	KP722022.1	China	2012	30-Jan-2015	I
HNX	KM189912.1	China	06-Aug-2012	12-Jul-2014	I
Hsd-12019	MT468550.1	China	2019	13-May-2020	I
JS-2012	KP257591.1	China	/	07-Dec-2014	I
JS-XJ5	OP512542.1	China	2015	22-Sep-2022	I
JY	KX880453.1	China	2012	19-Sep-2016	I
SD18	MT949536.1	China	Aug-2020	01-Sep-2020	I
SMX	KX880459.1	China	2014	19-Sep-2016	I
TJ	KJ789182.1	China	2012	05-May-2014	I
ZJ01	KM061380.1	China	10-Jan-2012	26-Jun-2014	I
DL14 08	KU360259.1	China	Aug-2014	28-Dec-2015	I
GD0304	MH582511.1	China	2015	02-Jul-2018	II
GY	KX880452.1	China	2012	19-Sep-2016	II
HB1201	KU057086.1	China	2012	05-Nov-2015	II
HeN1	KP098534.1	China	2012	04-Nov-2014	I
HLJ8	KT824771.1	China	2013	25-Sep-2015	II
HNXY	MN003371.1	China	22-May-2015	27-May-2019	II
HN-ZZ	MH321401.1	China	27-Sep-2017	07-May-2018	II
JS-2012 F50	MG551316.1	China	/	18-Nov-2017	II
JS-2012 F120	MG589642.1	China	/	28-Nov-2017	II
LC	MF434035.1	China	2015	03-Jul-2017	II
M5	KX880455.1	China	2012	19-Sep-2016	I
Namyangju	GQ325658.1	South Korea	1987	24-Jun-2009	II
qihe547	KT818618.1	China	2014	23-Sep-2015	I
TA2	MK622324.1	China	08-May-2017	12-Mar-2019	I
W-MPRV-2	MF940939.1	China	01-Sep-2014	11-Sep-2017	I
Xiang A	KP710981.1	China	Jun-2012	26-Jan-2015	I
B GII	AF257079.1	China	/	17-Apr-2000	I
FJ-2015	MF405136.1	China	Aug-2015	27-Jun-2017	I
FJ-YXJSJ2	MK610410.1	China	2019	04-Mar-2019	I
HBCL-2012	KX451219.1	China	24-Jan-2013	27-Jun-2016	I
HD2	MF409398.1	China	2017	28-Jun-2017	I

Supplementary Materials

A: ASFV

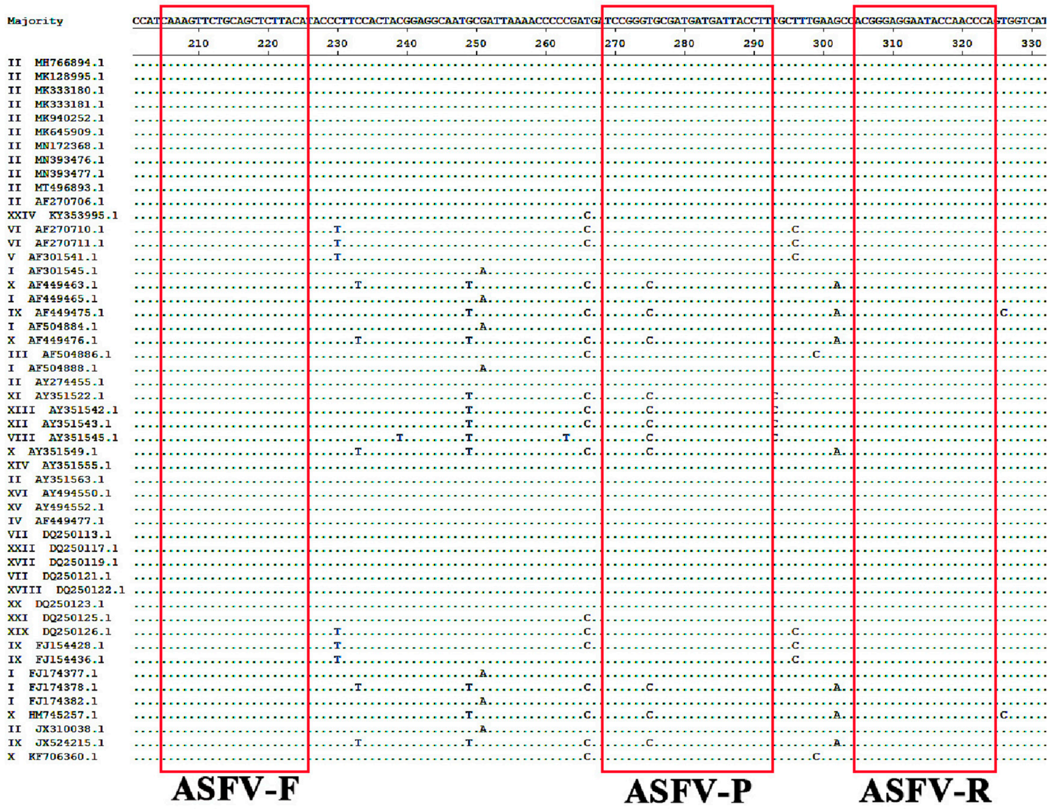

B: CSFV

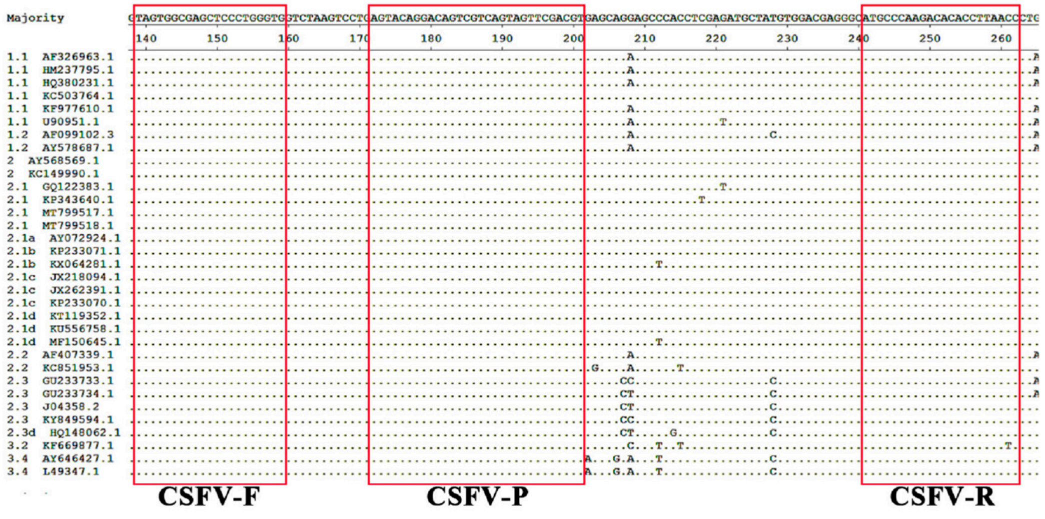

C: PRRSV

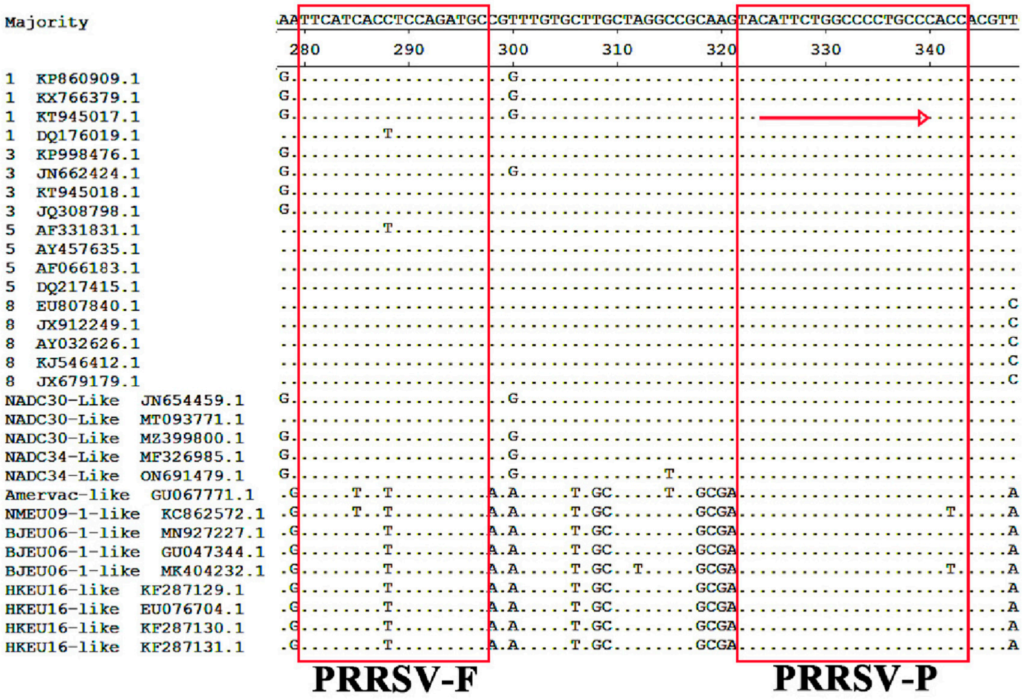

C: PRRSV

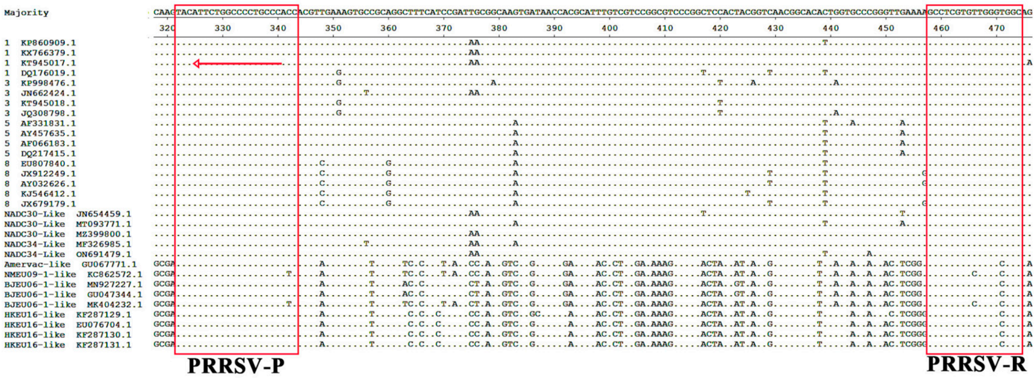

D: PRV

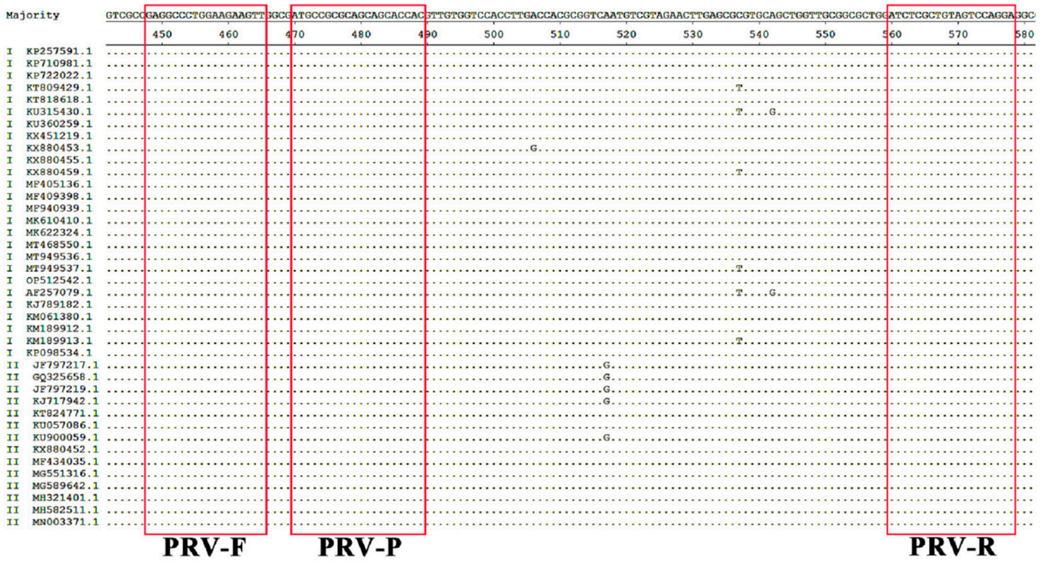

Figure S1. The multiple sequence alignments of ASFV (A), CSFV (B), PRRSV (C), and PRV (D). The forward (F)/ reverse (R) primers, and probe (P) are located in the conserved region of the target gene.
